# A rare association in a patient with non-muscle invasive bladder cancer: ureteral fibroepithelial polyp and ipsilateral renal cell carcinoma: a case report

**DOI:** 10.1186/s13256-021-03070-3

**Published:** 2021-09-26

**Authors:** Serkan Akan, Caner Ediz

**Affiliations:** 1 Department of Urology, University of Health Sciences, Fatih Sultan Mehmet Training and Research Hospital, Istanbul, Turkey; 2 Department of Urology, University of Health Sciences, Sultan Abdulhamid Han Training and Research Hospital, Tr- 34668 Istanbul, Turkey

**Keywords:** Fibroepithelial polyp, Renal cell carcinoma, Bladder cancer, Polyps, Case report

## Abstract

**Background:**

Fibroepithelial polyps located in the ureter constitute 2–6% of all benign tumors in the urinary system. Distinguishing these lesions from transitional cell carcinoma is essential to avoid unnecessary nephroureterectomy.

**Case presentation:**

A 59-year-old asymptomatic caucasian male patient was enrolled in follow-up for Ta low-grade transitional cell bladder cancer 4 years ago in our clinic. A suspicious, solid, contrast-enhancing mass 15 × 9 mm in diameter in the anteromedial mid-section of the left kidney, which was causing minimal washout and largely located in the parenchyma, was reported as renal cell carcinoma on computed tomography during routine controls. In the excretory phase, soft-tissue densities of approximately 30 mm in length, which were located in the distal part of the left ureter at a distance of 40 mm from the ureterovesical junction, extending towards the lumen suggested a urethral carcinoma. Urothelial lesion was reported as fibroepithelial polyp after histopathological examination. Partial nephrectomy for the mass, which was reported as renal cell carcinoma in the left kidney, was performed in the first postoperative month. Histopathological examination revealed Fuhrman grade 1 papillary type renal cell carcinoma. No recurrence was observed in the first year after treatment.

**Conclusions:**

Although our patient had a bladder transitional cell carcinoma and a suspicious renal cell carcinoma mass of 15 mm in the ipsilateral kidney, the patient was safeguarded from unnecessary nephroureterectomy early on by cross-sectional and endoscopic imaging of the ureter.

## Introduction

Fibroepithelial polyps (FEPs) of the ureter are benign tumors with a mesodermal origin. They represent 2–6% of all benign tumors in the urinary system [1]. Sepsis, immunosuppressant treatment and radiation therapy are in the differential diagnosis of FEPs or urological cancers [2]. Distinguishing these lesions from transitional cell carcinoma is essential to avoid unnecessary nephroureterectomy. However, half of the patients were treated with unnecessary ureterectomies in the literature. To the best of our knowledge, in the English literature, we present the first FEP case that is associated with renal cell carcinoma (RCC) and non-muscle invasive bladder cancer.

## Case report

A 59-year-old asymptomatic caucasian male patient was enrolled in follow-up for Ta low-grade transitional cell bladder cancer 4 years ago in our clinic. Intracavitary treatment was not performed, and no recurrence was observed last year during the follow-up period. The medical history of the patient included a smoking history of 30 years and no comorbidities other than hypertension. No additional findings were observed on physical examination, and all laboratory findings were within normal limits.

**Fig. 1:**
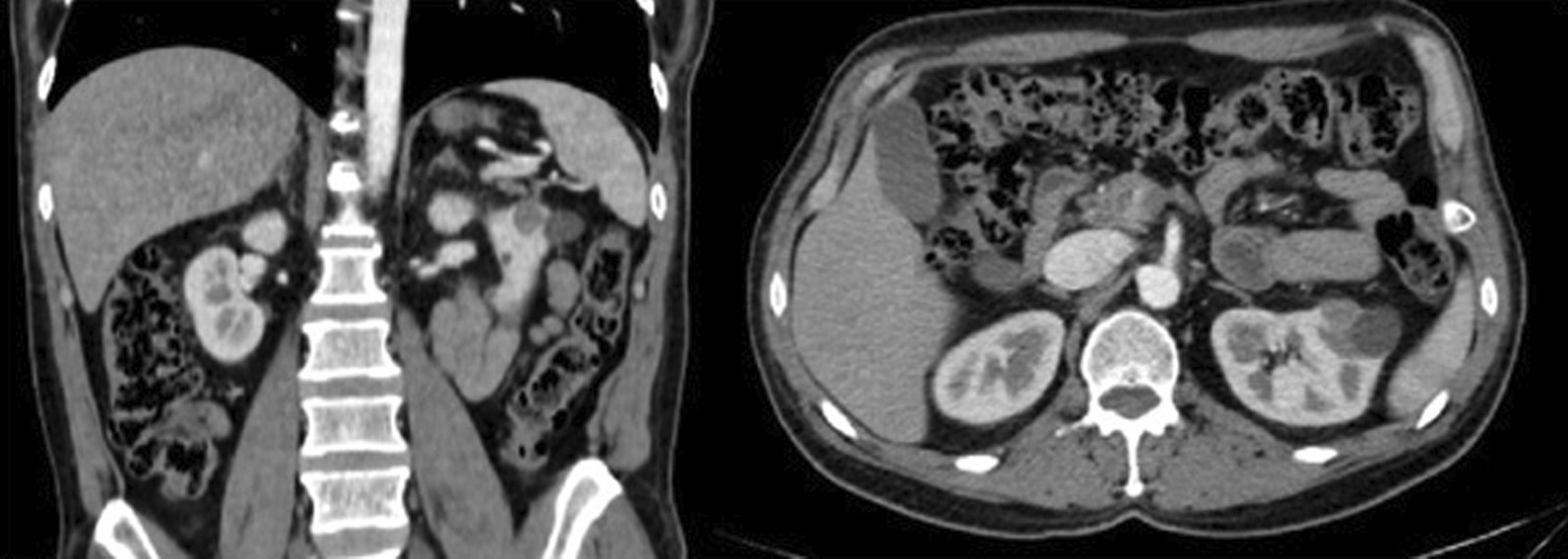
Coronal and transverse plane images of renal cell carcinoma in the left kidney

**Fig. 2:**
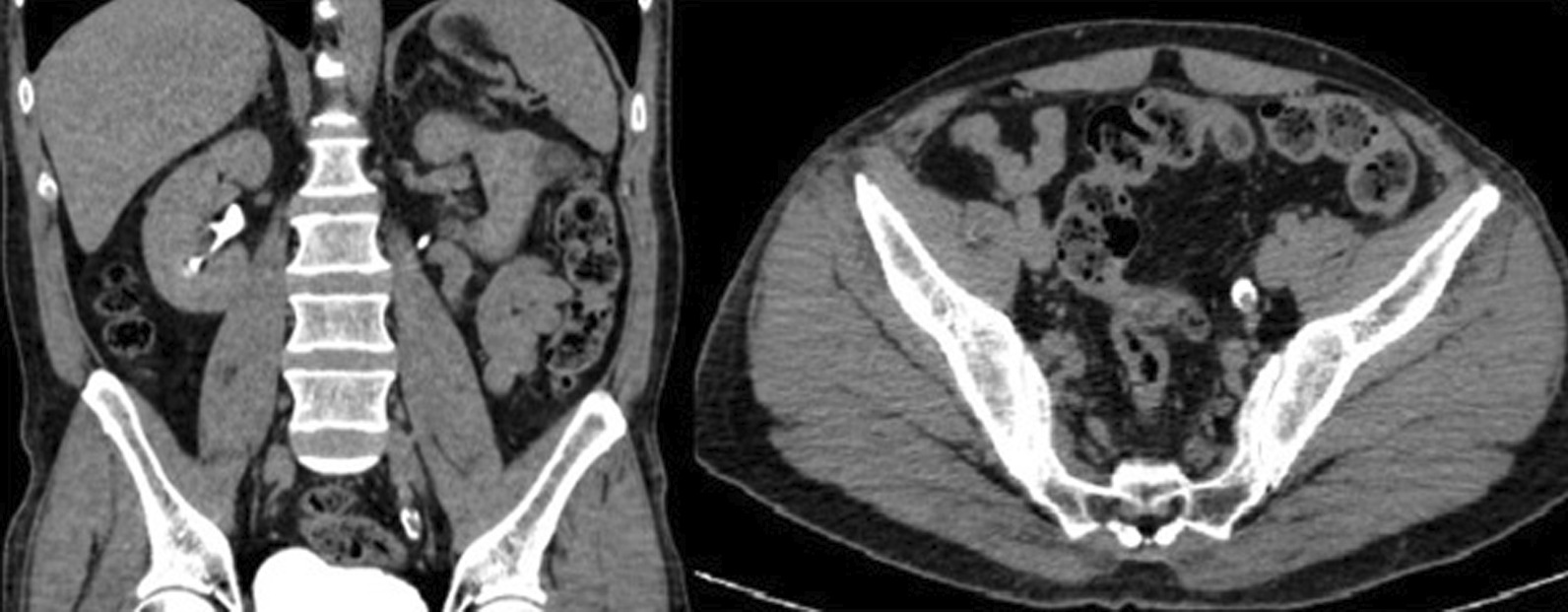
Filling defect in the urinary system in the excretory phase of triphasic dynamic abdominal computed tomography; coronal and transverse plane images of fibroepithelial polyps located in the left ureter

A suspicious, solid, contrast-enhancing mass 15 × 9 mm in diameter in the anteromedial mid-section of the left kidney, which was causing minimal washout and largely located in the parenchyma, was reported as renal cell carcinoma (RCC) in the arterial phase of triphasic contrast-enhanced computed tomography (CT) in the routine controls of the patient for transitional cell cancer (TCC) (Fig. 1). In the excretory phase, soft-tissue densities of approximately 30 mm in length, which were located in the distal part of the left ureter at a distance of 40 mm from the ureterovesical junction, extending towards the lumen suggested a urethral carcinoma (Fig. 2). No pathological finding was observed on thorax CT.

**Fig. 3:**
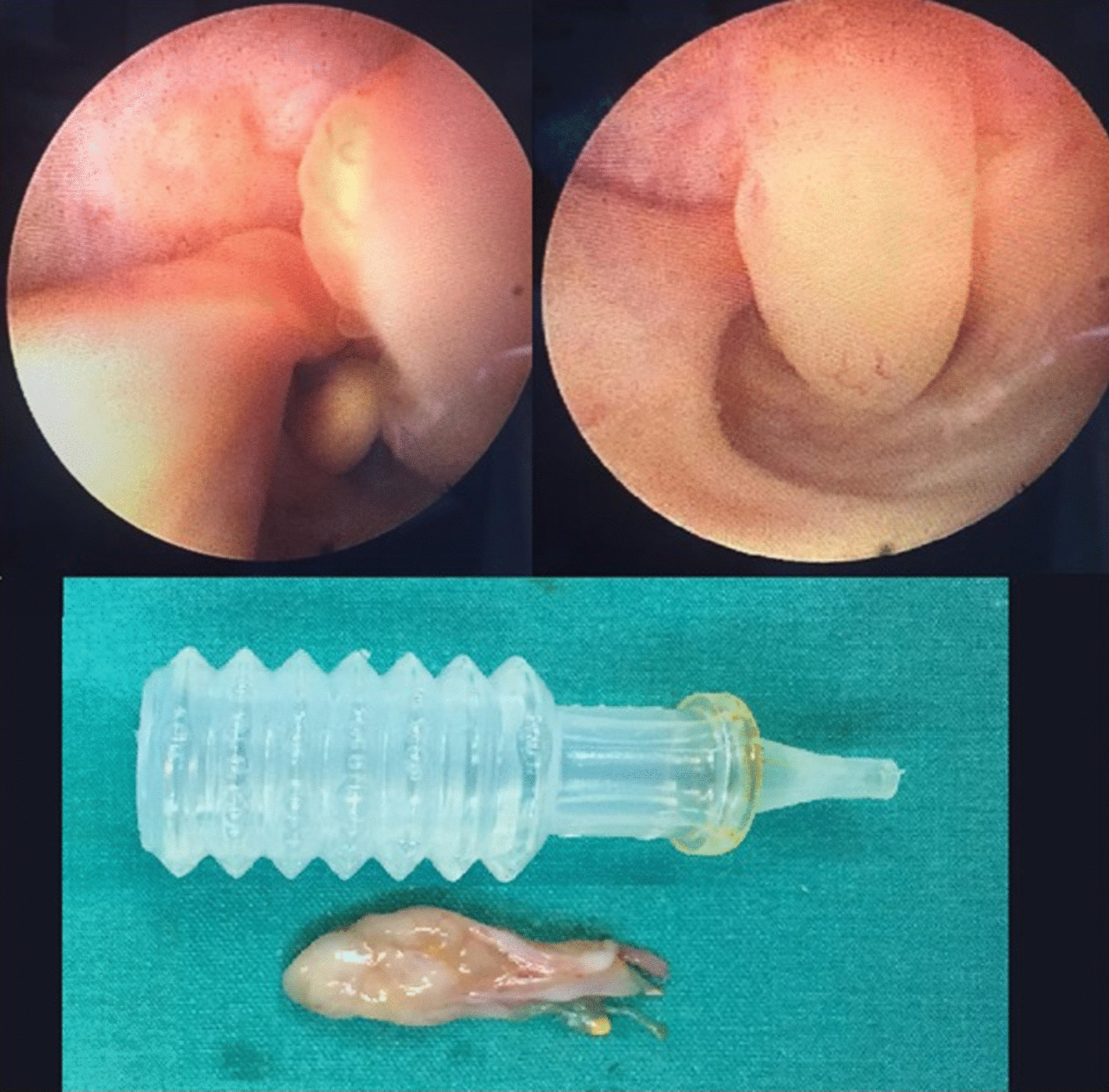
Appearance of ureteral fibroepithelial polyps and length of tissue

On endoscopic evaluation under general anesthesia, no suspicious formation was found in the urethra and bladder. On ureteroscopy performed in the same session, a polypoid-like tumoral formation with a pedicle at the base in the left distal ureter, approximately 25–30 mm in length, was observed (Fig. 3). The lesion was resected endoscopically following obtainment of a selective urine cytology sample from the ureter. To prevent a recurrence, the tumor base was ablated by a holmium:yttrium–aluminum–garnet (Ho:YAG) laser. Histopathological examination of the tumor revealed a FEP, and selective urine cytology was negative for malignant cells.

Partial nephrectomy for RCC mass in the left kidney was performed in the first postoperative month. Histopathological examination of the mass revealed a Fuhrman grade 1 papillary type RCC, and no recurrence was observed in the first year after treatment.

The patient was satisfied with the partial nephrectomy procedure, which somewhat preserved the kidney, and the treatment of the lesions in the bladder and ureter, which was performed without major surgery.

## Discussion

Benign tumors of the ureter are rare and constitute 20% of all ureteral tumors [3]. They can originate from epithelial or non-epithelial tissue. The most common non-epithelial benign tumor of the ureter is FEP [1]. Unlike squamous cell cancer or TCC originating from epithelium, non-epithelial ureteric tumors develop from mesenchymal tissues. Although the cause is not known exactly, it is thought that congenital lesions grow slowly or are secondary to inflammation, infection, or trauma. In our case, in addition to malignancies such as bladder and kidney cancer, the coexistence of a rare benign lesion such as FEP suggests genetic factors rather than secondary causes. However, the coexistence of multiple tumors is extremely rare, and it is unlikely that evidence-based information will be obtained to support this.

FEPs located in the ureter are usually diagnosed between the ages of 20 and 40 years and are more common in the ureteropelvic junction and upper ureter [4]. Intravenous urography and retrograde ureterography are the main diagnostic methods [5]. A filling defect is usually found in the urinary system in the excretory phase of triphasic dynamic contrast-enhanced CT, and urine cytology is typically negative. We present a patient with a FEP located at distal ureter at an advanced age, which is contrary to the current information. Triphasic abdominal CT was preferred for diagnosis. We think that triphasic dynamic contrast-enhanced CT is more effective than intravenous urography and retrograde ureterography owing to its evaluation of local invasion, regional lymph node involvement, or distant metastasis in the determination of suspicious lesions as benign or malignant.

The frequency of ureteral stones is increased (20.8%) in patients with FEP located in the ureter. Until today, coexistence with TCC has been reported in only two cases [6]. As far as we know, its coexistence with RCC has not been reported yet. Another important issue in this case report is that tumors were low-grade and non-aggressive in all cases. This may suggest that the etiologic factor is the same in the tumor formation.

Open-surgery, robot-assisted laparoscopic excision, laser ablation, and/or excision can be used in the treatment of FEP located in the ureter [7]. However, endoscopic resection is mostly sufficient [8]. The largest analysis in the literature regarding the diagnosis and treatment of FEP is a review article that includes 134 patients with ureteral FEP in 75 articles [9]. In this review, 43% of patients with FEP were treated with endoscopic resection, 23% of them with partial ureterectomy and 8.8% of them with nephroureterectomy. It is very important to distinguish these lesions from TCC to avoid unnecessary nephroureterectomies. Biopsy and histopathological examination are recommended to confirm the diagnosis of FEP before the definitive treatment [10]. In the first few years of the postoperative period, frequent urinalysis, ultrasonographic evaluation, intravenous urography, or abdominal CT are recommended in follow-up [11]. In our case, total excision of the lesion was endoscopically performed due to abdominal CT findings and intraoperative findings such as the appearance of the lesion that primarily suggested benign ureteral lesion. In the presence of suspicious lesions that preoperative and intraoperative findings cannot clearly define, intraoperative frozen section evaluation of the tissue sample can be performed. Fulgurization of the tumor base will minimize the possibility of recurrence at the expense of bleeding risk, and Ho: YAG laser may be preferred in terms of reduced ureteral stricture complication rates.

## Conclusion

Although FEPs are rare benign lesions of the ureter, half of the patients who were reported in the literature are treated with unnecessary ureterectomies. Although our patient had bladder transitional cell carcinoma and a suspicious RCC mass of 15 mm in the ipsilateral kidney, the patient was safeguarded from unnecessary nephroureterectomy early on by cross-sectional and endoscopic imaging of the ureter.

## Data Availability

Not applicable.

## Abbreviations

CT: Computed tomography
FEP: Fibroepithelial polyp
HoYAG: Holmium:yttrium–aluminum–garnet
RCC: Renal cell carcinoma
TCC: Transitional cell bladder cancer
